# Analysis of Cooperativity by Isothermal Titration Calorimetry

**DOI:** 10.3390/ijms10083457

**Published:** 2009-08-04

**Authors:** Alan Brown

**Affiliations:** Department of Biochemistry, University of Cambridge, 80 Tennis Court Road, Cambridge, UK; E-Mail: alan@cryst.bioc.cam.ac.uk

**Keywords:** isothermal titration calorimetry, stoichiometry, cooperativity, multiprotein complexes, thermodynamics, global analysis, NMR

## Abstract

Cooperative binding pervades Nature. This review discusses the use of isothermal titration calorimetry (ITC) in the identification and characterisation of cooperativity in biological interactions. ITC has broad scope in the analysis of cooperativity as it determines binding stiochiometries, affinities and thermodynamic parameters, including enthalpy and entropy in a single experiment. Examples from the literature are used to demonstrate the applicability of ITC in the characterisation of cooperative systems.

## 1. Introduction

Isothermal titration calorimetry (ITC) is a powerful and important technique for the study of the thermodynamics of macromolecular interactions. In an ITC experiment, two reactants are titrated against one another and the extent of binding is determined by direct measurement of heat exchange with the environment (a ubiquitous process in all biological interactions). It is the only technique where the binding constant (*K_b_*), Gibbs free energy of binding (Δ*G*), enthalpy (Δ*H*) and entropy (Δ*S*) can be directly and accurately determined from a single experiment [1]. Given the universal application of ITC and the ability to obtain full thermodynamic characterisation, the technique has found widespread applicability in the study of biological systems [2]. ITC also has the additional advantages that the experiments can be performed in a physiologically relevant buffer and the interacting species do not require immobilisation or chemical modification.

ITC also allows for an accurate determination of the reaction stoichiometry that is independent of the binding affinity. The reaction stoichiometry is determined from the titration equivalence point. Given the suitability of ITC to determine reaction stoichiometry, it is increasingly used in the analysis of systems that involve multiple binding events, such as the formation of multiprotein complexes [3] or the binding of multivalent ligands [4]. Systems that involve multiple binding events that occur at two or more interacting sites often demonstrate cooperativity, a mechanism of transferring information.

This review aims to illustrate the use of ITC in dissecting the thermodynamics of cooperativity, with examples taken from the literature. The use of correct experimental design and appropriate binding models are described as are recent advancements in fitting calorimetric data using global analysis and the synergy of ITC with NMR.

## 2. Selection of an Appropriate Binding Site Model

In a titration experiment, the ligand, *X*, in the syringe is added in small aliquots to the macromolecule, *M*, in the calorimeter cell. At the beginning of the experiment the calorimetric cell is filled with the macromolecule, with an effective volume (*V*_0_) that is sensed calorimetrically. As the titration proceeds, each injection drives a volume of liquid out of the calorimetric cell that is equal to the injection volume, υ. Thus the concentration of the macromolecule decreases slightly after each injection. For analysis of the isotherms, it is necessary to correct for this displacement; the total concentrations of ligand, [*X*]*_t_*, and macromolecule, [*M*]*_t_*, in the calorimetic cell after each injection *i* are given by:

(1) $${\left[X\right]}_{t,i}=\;{\left[X\right]}_{0}\;\left(1-{\left(1-\frac{\upsilon }{V_{0}}\right)}^{i}\right),{\left[M\right]}_{t,i}={\left[M\right]}_{0}{\left(1-\frac{\upsilon }{V_{0}}\right)}^{i}$$

where [*M*]_0_ and [*X*]_0_ are the initial concentrations of the macromolecule and the ligand, respectively. After each injection, a number of molecules of ligand have been added to a total amount of macromolecule [*M*]*_t_* in the cell. As a result of the binding, the concentrations the of free ligand [*X*] and the complex [*MX*] change. The interaction is accompanied by heat exchange, measured by the instrument as the necessary energy to maintain a constant temperature (in microcalories per second).

The heat after each injection is derived by calculating the area under each peak. The size of the heat event is directly proportional to the amount of binding that occurs. As the macromolecule become saturated with the ligand, the magnitudes of the peaks decrease until the peak size reflects dilution and mechanical effects, resulting in a classical sigmoidal curve. The total heat content, *Q*, of the solution contained in *V*_0_ is given by:

(2) $$Q={\left[M\right]}_{t}V_{0}n\Delta H\Theta$$

where *n* is the number of binding sites per macromolecule, Δ*H* is the enthalpy of binding and Θ is the fraction of sites occupied by ligand *X*.

The successful extraction of thermodynamic parameters relies on the use of nonlinear least squares curve fitting while employing an appropriate model that describes the interaction under study. The simplest model is one where there is a single independent binding site, forming a 1:1 ligand/macromolecule complex (Equation 3).

(3) M+X⇌ MX

The aim of the fitting procedure is to find those values of parameters which best describe the data. The fitting process is typically undertaken using a one-site model based on the Wiseman isotherm [5, 6];

(4) $$Q=\frac{n{\left[M\right]}_{t}V_{0}\Delta H}{2}\left[1+\frac{{\left[X\right]}_{t}}{n{\left[M\right]}_{t}}+\frac{1}{c}-\sqrt{{\left(1+\frac{{\left[X\right]}_{t}}{n{\left[M\right]}_{t}}+\frac{1}{c}\right)}^{2}-\frac{4{\left[X\right]}_{t}}{n{\left[M\right]}_{t}}}\right]$$

where *c* is a unitless parameter defined in Equation 5, *K_b_* is the binding constant and *K_d_* is the dissociation constant (1/*K_b_*).

(5) $$c=nK_{b}{\left[M\right]}_{t}=n\frac{{\left[M\right]}_{t}}{K_{d}}$$

The shape of the isotherm varies according to the *c* value. It is only in *c* value ranges of approximately 1 to 1000 that isotherms can be accurately deconvoluted [5]. However, for low-affinity systems, it is not always possible to achieve *c* values in the preferred range due to limited receptor and/or ligand solubility. Whilst theoretical and experimental evidence supports extending the experimental window for ITC to much lower values of *c* to determine Δ*H* and *K_b_*, it requires *n* to be fixed and accurately known [6]. Assuming the experiment is designed to ensure the *c* value falls in the permitted range, it is possible to use *n* as a floating parameter.

Therefore, in a carefully designed ITC experiment, nonlinear least-squares fitting to the binding data using the one-site model determines the values of *n*, *K_b_* and Δ*H*. A full thermodynamic profile is then obtained using the relationships shown in Equation 6.

(6) $$\Delta G^{{}^{\circ}}=\Delta H^{{}^{\circ}}-T\Delta S^{{}^{\circ}}=-RT\;\text{ln}\;K_{b}$$

where Δ*G*°, Δ*H*° and Δ*S*° are the Gibbs free energy, enthalpy and entropy of binding, respectively. *T* is the absolute temperature and *R* = 1.98 cal mol^−1^ K^−1^ is the ideal gas law constant [5].

However, in order to study the complex macromolecular interactions that display cooperativity, it is necessary to use alternative binding models that have been developed to take into account multiple binding sites and the possible cooperativity between the binding sites. Also, when studying complex macromolecular interactions, a single ITC experiment is often insufficient to sample the shape of the binding isotherm and may not allow derivation of the binding and cooperativity parameters. For complex interactions to be accessible to ITC analysis, multiple titration experiments need to be performed in which the contents of the syringe and calorimetric cell are varied, so that the shape of the isotherm can be fully explored.

## 3. Multiple Binding Sites

In a 1:1 interaction (Equation 3) there is only one association constant. However, the equilibrium of a macromolecule with *n* multiple ligand binding sites can be described by two different association constants; the macroscopic and microscopic association constants. The macroscopic association constant is model independent and describes the overall behaviour of the *n* sites, whereas the microscopic association constant, *k*, takes into account how binding occurs at each site and is therefore model dependent [7]. Macroscopic association constants are determined by ITC and can take the form of either an overall binding constant, *β_j_*, or a stepwise binding constant, *K_j_*, for ligation of the *j*th site.

(7) $$M+\;jX⇌\;MX_{j},\;\beta_{j}=\frac{\left[MX_{j}\right]}{\left[M\right]{\left[X\right]}^{j}}$$

(8) $$M+X_{j-1}⇌MX_{j},\;K_{j}=\frac{\left[MX_{j}\right]}{\left[MX_{j-1}\right]\left[X\right]}$$

where *j* is any integer between 0 and *n*. The stepwise and overall association constants are related and can be transformed into one another using the relationships shown in Equation 9.

(9) $$\beta_{j}=\prod_{i=1}^{j}K_{i},\;K_{j}=\frac{\beta_{j}}{\beta_{j-1}}$$

As the titration of multivalent macromolecule with ligand proceeds, the average number of ligand molecules bound per macromolecule, *ν*, increases. *ν* can be calculated by Equation 11, and can take values between 0 and *n*.

(10) $$\nu \;=\frac{{\left[X\right]}_{b}}{{\left[M\right]}_{t}}=\frac{\sum_{j=0}^{n}j\left[MX_{j}\right]}{\sum_{j=0}^{n}\left[MX_{j}\right]}=\frac{\left[MX\right]+2\left[MX_{2}\right]+3\left[MX_{3}\right]+\ldots }{\left[M\right]+\left[MX\right]+\left[MX_{2}\right]+\left[MX_{3}\right]+\ldots }$$

(11) $$\nu =\frac{\sum_{j=0}^{n}jK_{j}{\left[X\right]}^{j}}{{\sum_{j=0}^{n}K_{j}\left[X\right]}^{j}}$$

## 4. Cooperativity

Biological systems are complex networks that require careful regulation [8]. Cooperativity is an effective mechanism of regulation [9]. It provides a medium to transfer information, amplify or nullify a response to changes in local concentration and regulate the overall reaction pathway. Cooperativity is a hallmark of the mode of assembly and activity of macromolecular complexes. Cooperative effects are either positive (synergistic) or negative (interfering), depending on whether the binding of the first ligand increases or decreases the affinity for subsequent ligands. Noncooperative (additive) binding does not affect the affinity for subsequent ligands and the interaction sites can be considered independent.

**Figure 1.**
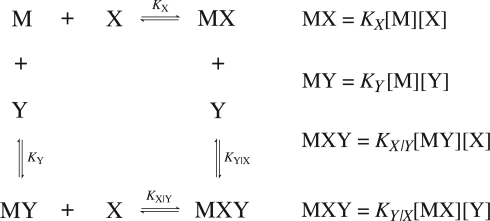
Reaction scheme for the binding of heterogeneous ligands, *X* and *Y*, to a macromolecule, *M*, containing two binding sites. The stepwise association constants, *K_X_* and *K_Y_*, for the binding of ligands *X* and *Y* respectively, to free macromolecule are shown, as the association constants for the binding of ligands to preformed complexes, *K_X_*_|_*_Y_* and *K_Y|X_*.

Consider a common case of cooperativity where one macromolecule, *M*, is capable of binding two ligand molecules, *X* and *Y*. The general binding scheme is shown in Figure 1.

If the binding is regulated by cooperativity, in that the binding of one ligand influences the binding of the second, then the association constants will differ by a unitless term defined as the cooperativity constant, *α*. Therefore, it is necessary to distinguish between the association constants for the binding of the free ligands (*K_X_* and *K_Y_*) from the association constants for the binding of the second ligand given that the first is already bound (*K_Y|X_* and *K_X|Y_*). The value of the cooperativity constant, *α*, indicates whether the formation of a higher order complex is negatively (*α* *<* 1), positively (*α* *>* 1) or non (*α* = 1) cooperative. Negative values of *α* are not permitted. The relationship between the individual association constants, the association constants after initial binding and the cooperativity constant are shown in Equation 12. The binding scheme shown in Figure 1 and the relationships in Equation 12 hold true for homotrophic ligands, where ligand *Y* is substituted with *X′* (denoting a second ligand molecule).

(12) $$\begin{array}{l} K_{Y|X}=\alpha K_{X}\\ K_{X|Y}=\alpha K_{Y} \end{array}$$

Even for a macromolecule with just two binding sites there are at least six possible binding mechanisms. The binding sites may be; identical, but independent (*α* = 1); identical with negative cooperativity (*α* *<* 1); identical with positive cooperativity (*α* *>* 1); or nonidentical with neutral, negative or positive cooperativity.

## 5. Cooperativity: Thermodynamics and Conformational Changes

Cooperativity is often ascribed to conformational changes in macromolecular structure. However, it has been demonstrated that cooperative processes need not involve large conformation changes, but can by transmitted through subtle changes in protein motions [10]. Proteins are dynamic ensembles of conformations [11] in which allosteric motions occur even in the absence of ligand. Ligand-binding merely shifts the dynamic equilibrium by preferentially stabilising a particular motion. Changes in free energies of a few kcal mol^−1^ can be easily achieved by a slight stiffening of a few of the many global dynamic modes of motion available to a protein [12]. It is therefore preferable to describe cooperativity both in terms of conformational changes (if observable) and thermodynamics as cooperativity is fundamentally thermodynamic in nature.

ITC allows full thermodynamic characterisation of both the global (Equation 6) and cooperative processes. The enthalpy term, simplistically, reflects reinforcement of the interactions between the ligand and the receptor. These include functional group interactions (ionic, hydrogen bonds, van der Waals interactions), conformational changes, polarisation of the interacting groups and electrostatic complementarity. The simplest description of entropy is that it is a measure of disorder in a system. Changes in the binding entropy reflect loss of motion caused by changes in internal rotations and vibrations of the molecules. Desolvation and the release of counterions upon complex formation can contribute significantly to the entropy term [13]. The enthalpy and entropy terms are intimately related. For example, an increase in enthalpy by tighter binding can have a direct effect on the entropy by loss of mobility of the molecules involved in the interaction. This phenomenon of entropy-enthalpy compensation is widely observed, but its relevance is hotly debated [14, 15].

In cases where the compensation of enthalpy and entropy is imprecise, then an increase in enthalpy can lead to a favourable contribution to the Gibbs free energy of binding, which has been termed the enthalpic chelate effect [16]. In a weakly associated complex, there are large intermolecular motions that weakens the enthalpy. Binding of additional ligands restricts protein intramolecular motion, through a process known as “structural tightening” [17]. Reduced flexibility of the binding sites means that all individual interactions display a more favourable enthalpy term. This differs from the classical entropic chelate effect where the entropic penalty associated with forming a biomolecular complex is removed by the first interaction so that all subsequent interactions are therefore enhanced.

Continuing the above example of heterotropic binding to a macromolecule with two dependent binding sites, the Gibbs free energy associated with the formation of each complex can be determined from:

(13) $$\Delta G_{X}=-RT\text{ln}K_{X}$$

(14) $$\Delta G_{Y}=-RT\text{ln}K_{Y}$$

(15) $$\Delta G_{XY}=-RT\text{ln}\;\left(\alpha K_{X}K_{Y}\right)=\Delta G_{X}+\Delta G_{Y}+\Delta g$$

The cooperativity constant is a true equilibrium constant and is related to the interaction or cooperativity Gibbs free energy, enthalpy, and entropy by Equations 16–18, as obtained by applying the Gibbs–Helmholtz relationship, which is described in more detail by Velázquez-Campoy [18].

(16) $$\Delta_{g}=-RT\text{ln}\alpha =\Delta G_{XY}-\Delta G_{X}-\Delta G_{Y}$$

(17) $$\Delta h=RT^{2}{\left(\frac{\partial \text{ln}\alpha }{\partial T}\right)}_{P}=\Delta H_{XY}-\Delta H_{X}-\Delta H_{Y}$$

(18) $$\Delta s=R\left(\text{ln}\alpha +T{\left(\frac{\partial \text{ln}\alpha }{\partial T}\right)}_{P}\right)=\Delta S_{XY}-\Delta S_{X}-\Delta S_{Y}$$

Using these thermodynamic descriptors, three types of cooperativity have been defined; type I, II and III. Type I is governed by entropy, type II is governed by both entropy and enthalpy, and type III is predominantly enthalpic [19]. Positive cooperativity can be both enthalpy- and entropy-driven. Entropy-driven positive cooperativity occurs when the combined entropic cost of the sequential binding events is lower than the summation of two independent events [20, 21]. Enthalpy-driven positive cooperative occurs when binding of the first ligand results in a conformational change at the second binding site, rendering it higher affinity to the ligand [22]. On the other hand, published examples of negative cooperativity tend to be mainly entropy-driven and occur when binding results in a loss of configurational entropy. This is the case for the homodimeric enzyme glycerol-3-phosphate:CTP transferase, where NMR studies showed that the strong negative cooperativity between the first and second binding of its substrate, CTP, was due to the loss of configuration entropy of the protein backbone [23]. Entropy-driven negative cooperativity is also observed in the binding of multivalent carbohydrates to legume lectins [24]. Enthalpy-driven negative cooperativity has been described in cases where ligand binding leads to a conformational change that results in the dissociation of a complex, e.g., in the dissociation of the trimeric G-protein following activation of the seven-helix receptor family [25].

## 6. Reverse ITC Experiments

When macromolecules with multiple binding sites are titrated with ligands (or conversely, macromolecules with one site are titrated with multivalent ligands), the binding curve represents a description of the global energetics of the multisite system and may display multiple phases. The shape of the isotherm can differ significantly depending on whether the macromolecule or ligand is in the calorimetric cell. This is especially the case when multivalent systems display positive cooperativity and the intermediate state can be poorly populated. The isotherm is dominated by unsaturated and fully saturated states, with few singly-bound states, often giving a rather featureless binding curve [26].

In order to fully resolve the binding and cooperative thermodynamics, it is necessary to perform a reverse titration, where the titrant and titrand orientations are reversed. Reverse titrations should be conducted to check the stoichiometry and the suitability of the binding model [27]. For 1:1 biomolecular reactions it is expected that the measured thermodynamic parameters are invariant when changing the orientation of the experiment. However, this is rarely the case as one species may display greater aggregation when concentrated.

In cases where normal and reverse titrations are insufficient to fully describe the microscopic binding constants, it may be necessary to attempt global fitting analysis or combine the ITC data with other biophysical data that can explore cooperativity, such as NMR (see below) and spectrofluorometry.

## 7. General Analysis Procedure

Suitable mathematical methods for analysing cooperative ITC data will now be described. These methods are either model independent (the binding polynomial) or model dependent, where the general binding mechanism under study is known. However, for all techniques, the general analysis procedure is very similar and can be thought to consist of six steps; (1) selection of an appropriate model/binding polynomial; (2) calculation of the total macromolecule and ligand concentrations for each injections using Equation 1; (3) solvation of the the ligand conservation equation for each experimental point assuming certain values; (4) calculation of the concentrations of each different complex or bound state; (5) calculation of the expected signal, assuming certain values for the binding enthalpies; and (6) obtaining the optimal constants and enthalpies that reproduce the experimental data using an iterative method, *i.e.*, nonlinear least squares regression.

## 8. Analysis of Cooperativity Using the Binding Polynomial

Under equilibrium conditions, the binding of ligand by a multivalent macromolecule may be described by a binding polynomial [28]. The use of the binding polynomial as a general method to analyse ITC data has been described in detail by Freire *et al.* [29]. It has a major advantage in that it is model independent, but does require the number of binding sites to be known or fixed prior to analysis. Data analysis utilising binding polynomials yields macroscopic association constants and enthalpy values. These values can be immediately translated into the model specific constants once the correct model is determined. In the absence of a validated binding model, the binding polynomial should be the preferred starting point for data analysis.

The binding polynomial is defined as the partition function, *P*, of the system. It is provided by the summation of the different concentrations of bound species relative to the free macromolecule concentration, or alternatively, as the summation of the concentration of free ligand in terms of the macroscopic association constant:

(19) $$P=\sum_{j=0}^{n}\frac{\left[MX_{j}\right]}{\left[M\right]}=\sum_{j=0}^{n}K_{j}{\left[X\right]}^{j}$$

From the partition function, the fraction or population of each species *F_j_*, the average number of ligand molecules bound per macromolecule *ν*, the average excess molar enthalpy 〈Δ*H*〉 and the average Gibbs free energy 〈Δ*G*〉 can be obtained:

(20) $$F_{j}=\frac{\left[MX_{j}\right]}{{\left[M\right]}_{t}}=\frac{K_{j}{\left[X\right]}^{j}}{P}$$

(21) $$\langle \Delta G\rangle =-RT\text{ln}\left(\sum_{j=0}^{n}\beta_{j}{\left[X\right]}^{j}\right)$$

(22) $$\langle \Delta H\rangle =\frac{\sum_{j=0}^{n}\beta_{j}{\left[X\right]}^{j}\Delta H_{j}}{P}=\sum_{j=0}^{n}F_{j}\Delta H_{j}$$

(23) $$\nu =\frac{\sum_{j=0}^{n}\beta_{j}{\left[X\right]}^{j}j}{P}=\sum_{j=0}^{n}F_{j}j=\langle j\rangle$$

The use of the binding polynomial in the analysis of ITC is, perhaps, best illustrated through the use of an example. Consider a macromolecule with two binding sites for a homotropic ligand. The occupancy of the binding sites depends on the ligand concentration, the association constants and the presence or absence of a cooperativity factor. Calculation of the relative concentrations in each state depending on the binding model is given in Table 1.

Binding polynomials for each model can be obtained by the summation of the relative concentrations for each state in that model, Equation 19. The general binding scheme, however, is model independent as it is only concerned with the macroscopic association constants. The general binding polynomial for a two-site system is given by:

(24) $$P=1+\beta_{1}\left[X\right]+\beta_{2}{\left[X\right]}^{2}$$

The binding polynomial acts as the starting point for analysis of ITC data. Firstly, the total ligand concentration, [*X*]*_t_*, is expressed as the sum of free and bound ligand, Equation 25. The total ligand and macromolecular concentrations (after correction for liquid displacement from the calorimetric cell, Equation 1 are known. The values of the macroscopic association constants will determine the concentrations of both free ligand and formed complexes.

(25) $${\left[X\right]}_{t}=\left[X\right]+{\left[X\right]}_{b}=\left[X\right]+{\left[M\right]}_{t}\nu =\left[X\right]+{\left[M\right]}_{t}\frac{\partial \text{ln}P}{\partial \text{ln}\left[X\right]}$$

The values of the macroscopic association constants (*β_j_*) and binding enthalpies (Δ*H_j_*) are obtained through nonlinear least squares regression analysis of the experimentally-determined heat event associated with each injection:

(26) $$q_{i}=V_{0}\left({\left[M\right]}_{t,i}{\langle \Delta H\rangle }_{i}-\left(1-\frac{\upsilon }{V_{0}}\right){\left[M\right]}_{t,i-1}{\langle \Delta H\rangle }_{i-1}\right)$$

(27) $$=V_{0}\sum_{j=1}^{n}\Delta H_{j}\left({\left[MX_{j}\right]}_{i}-\left(1-\frac{\upsilon }{V_{0}}\right){\left[MX_{j}\right]}_{i-1}\right)$$

Thus, analysis of the ITC data provides accurate values for *β_j_* and Δ*H_j_*. Once these optimal values have been calculated, it is possible to determine the thermodynamic parameters using the relationships shown in Equations 21–23. It is also possible to determine the cooperativity factor, *α*, by Equation 28. The value of the cooperativity parameter provides a very strong indication of the true binding model. It is then possible to relate the macroscopic binding parameters to the microscopic binding parameters, *k_j_* and Δ*h_j_* [29].

(28) $$\alpha =\frac{4\beta_{2}}{\beta_{1}^{2}}$$

## 9. Heterotropic Interactions

For cases where two different ligands bind a macromolecule, cooperativity may result through three mechanisms; (1) both ligands bind to the same binding site; (2) both ligands bind to sites very close to one another, so that the ligands themselves or binding site residues in the macromolecule interact; and (3) both ligands bind to binding sites distant to one another, but are coupled through a change in protein dynamics/conformation. An exact analysis has been developed that determines the thermodynamic parameters for cooperative binding with heterotropic ligands [18].

Consider the titration of ligand *X* into a calorimetric cell containing both macromolecule, [*M*]*_t_* and ligand [*Y*]*_t_*. For data analysis, the total concentration of ligand *Y* in calorimetric cell needs to be adjusted for displacement in the same way the macromolecule concentration is handled, see Equation 1. The macromolecular concentrations can then be written as a set of nonlinear equations:

(29) $${\left[M\right]}_{t}=\left[M\right]+k_{X}\left[M\right]\left[X\right]+k_{Y}\left[M\right]\left[Y\right]+\alpha k_{X}k_{Y}\left[M\right]\left[X\right]\left[Y\right]$$

(30) $${\left[X\right]}_{t}=\left[X\right]+k_{X}\left[M\right]\left[X\right]+\alpha k_{X}k_{Y}\left[M\right]\left[X\right]\left[Y\right]$$

(31) $${\left[Y\right]}_{t}=\left[X\right]+k_{Y}\left[M\right]\left[Y\right]+\alpha k_{X}k_{Y}\left[M\right]\left[X\right]\left[Y\right]$$

Assuming values of the association and cooperativity constants it is possible to solve the set of nonlinear equations numerically by the Newton-Rhapson method giving the free concentrations of the reactants, [*M*], [*X*] and [*Y*]. Once the free concentrations are known, the concentrations of the complexes, [*MX*], [*MY*] and [*MXY*] can be determined by applying the mass action law, shown in Figure 1. Once the values have been determined, it is possible to evaluate the heat effect, *q_i_*, associated with each injection by nonlinear least squares fitting;

(32) $$\begin{array}{l} q_{i}=\;V_{0}(\Delta H_{X}\left({\left[MY\right]}_{i}-\left(1-\frac{\upsilon }{V_{0}}\right){\left[MX\right]}_{i-1}\right)\\ \;\;\;\;\;\;\;\;\;+\Delta H_{Y}\left({\left[MY\right]}_{i}-\left(1-\frac{\upsilon }{V_{0}}\right){\left[MX\right]}_{i-1}\right)\\ \;\;\;\;\;\;\;\;+\left(\Delta H_{X}+\Delta H_{Y}+\Delta h\right)\left({\left[MXY\right]}_{i}-\left(1-\frac{\upsilon }{V_{0}}\right){\left[MXY\right]}_{i-1}\right)) \end{array}$$

The usefulness of this exact method is that only one titration experiment is required to determine the interaction parameters instead of a series of experiments, saving both time and material. Material can be significant in the study of multicomponent complexes where each experiment uses at least three species.

### 9.1. Example: Ferredoxin:NADP^+^ Reductase

In *Anabaena* PCC 7119, the flavoenzyme FNR catalyses electron transfer from a ferredoxin electron donor protein (Fd) to a single NADP^+^ molecule through the formation of a transient ternary complex. The crystal structure of the 1:1 complex between Fd and FNR-NADP^+^ has been solved [30]. The redox centers of the two molecules are in close proximity, approximately 6 Å, to allow direct electron transfer. Comparison with the structure of free FNR revealed that binding Fd caused a notable conformational change in a loop region of FNR that permitted additional interactions with the Fd molecule.

**Figure 2.**
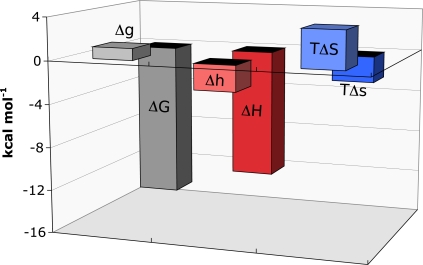
Global and cooperative thermodynamic parameters associated with the negatively cooperative binding of Fd to FNR-NADP^+^.

Titrations of FNR-NADP^+^ complex with Fd were performed and the data analysed using the binding formalism for heterotropic interactions [18, 31]. At pH 8.0, Fd bound FNR-NADP^+^ with a cooperativity constant, *α* of 0.17 indicating that the binding affinity is reduced by sixfold when NADP^+^ is prebound to FNR. The thermodynamic parameters for the binding of Fd to FNR-NADP^+^ are shown in Figure 2. The influence of NADP^+^ causes strong negative cooperativity, corresponding to a Δ*G* of 1.1 kcal mol^−1^. The cooperativity enthalpy is favourable (Δ*h* of −2.4 kcal mol^−1^), whereas the entropy is unfavourable (-*T*Δ*s* of 3.5 kcal mol^−1^). It is postulated that conformational changes that occur as a result of NADP^+^ being present regulates the cooperativity.

## 10. Cooperativity of Long-Chain Macromolecules with Multiple Binding Sites

**Figure 3.**
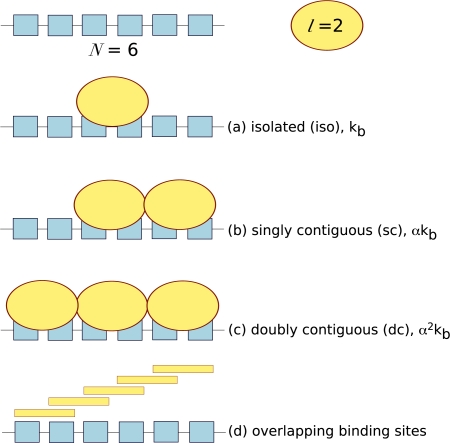
The three distinguishable types of ligand binding sites: isolated (iso), singly contiguous (sc) and doubly contiguous (dc). The potential number of binding sites is given by (*N* − *l* + 1), so that in this example where *N* = 6 and *l* = 2, there are five potential binding sites.

ITC is often used to measure the interactions between ligands and long-chain macromolecules (often considered one-dimensional lattices) such as nucleic acids and carbohydrates. These long-chain macromolecules consist of repeating units that form a number of potential binding sites (*N*) distributed along the molecule. Each ligand has a particular footprint: the minimal number of repeating units necessary to support binding (*l*). The number of ligand molecules bound per macromolecule (*ν*) is defined as:

(33) $$\nu =\frac{{\left[X\right]}_{b}}{{\left[M\right]}_{t}}$$

where [*X*]*_b_* is the concentration of bound ligand, and [*M*]*_t_* is the total concentration of macromolecule. In the characterisation of protein-lattice systems, one often wants to determine the affinity of the interaction and how the affinity varies with lattice heterogeneity, the binding site size (*l*) and whether ligand binding is cooperative. ITC provides an ideal platform to answer these questions, however it has often been neglected and few examples exist in the literature. Lattice systems require a different type of theoretical analysis to take into consideration potential binding site overlap and the cooperativity between neighbouring ligands. Ligands can bind to lattices in three ways: isolated binding, where ligand binds in the absence of a neighbouring ligand; singly contiguous binding, where the ligand binds and interacts with an adjacent ligand; and doubly contiguous binding, where the ligand binds with two flanking ligands. In isolated binding the affinity of the ligand for the macromolecule reflects the intrinsic association constant, whereas in the presence of neighbouring ligands the cooperativity factor, *α* needs to be accounted for, see Figure 3.

Also, in a homogenous lattice with no bound ligand, any particular residue can potentially initiate a ligand binding site. Thus, the actual number of free ligand binding sites on an unoccupied lattice is (*N* − *l* + 1). For example, a macromolecule of six repeating units (*N* = 6) and ligand with a footprint of two units (*l* = 2) will have five potential overlapping binding sites, Figure 3.

As shown previously, transformation of binding data to a linear representation can facilitate data analysis. Therefore in a transformation to a Scatchard plot *ν*/[*X*] is represented as a function of *ν*:

(34) $$\frac{\nu }{\left[X\right]}=f\left(\nu ;N,l,k,\alpha \right)$$

where *k* is the ligand dissociation constant and *α* is the cooperativity factor. For interactions that do not display cooperativity, the *α* term can be omitted. The Scatchard plot was originally derived for interactions of small molecules with multiple but discrete and isolated binding sites on proteins [32]. Thus, the plot is only linear when *l* = 1, em i.e., when the ligand footprint is a single nucleotide base or a monosaccharide unit, and the binding sites are equivalent and independent. This is rarely the case. In 1974, McGhee and von Hippel derived a closed form of the Scatchard representation that is valid for any size of ligand footprint and takes into consideration cooperative interactions between contiguously-bound ligands [33]. This model is widely acknowledged to be appropriate for both cooperative and non-cooperative phenomena. Implementation of the McGhee–von Hippel formalism in the interpretation of ITC binding data has been presented in detail by Velázquez-Campoy [34] and is summarised here.

Firstly, consider a long-chain molecule with homogenous non-cooperative ligand binding sites. In this instance, it is not necessary to take into account a cooperativity factor:

(35) $$\frac{\nu }{\left[X\right]}=\frac{N-l\nu }{k}{\left(\frac{N-l\nu }{N-\left(l-1\right)v)}\right)}^{i-1}$$

This model assumes an infinite polymer. ITC experiments will generally be performed with polymers of known finite length and end effects, *i.e.*, those from the reducing and non-reducing ends of a polysaccharide will have an effect on binding. The McGhee–von Hippel formalism has been extended by Tosodikov *et al.* for finite lattices by incorporating end effects [35], but the original model has been shown to be a reasonable approximation where *N*/*l* is significantly lower than 30 [36]. As previously mentioned, the Scatchard plot is only linear in the special case where *l* = 1. When *l >* 1, the Scatchard plot demonstrates positive curvature reflecting the entropic resistance to saturation, *i.e.*, as binding proceeds to saturation it becomes more difficult to find *l* unoccupied binding residues. The larger the value of *l*, the larger this effect becomes.

In practice, to solve this equation using the information present in the ITC experiment, it is necessary to express [*X*] in terms of the total concentration of ligand and macromolecule ([*X*] = [*X*]*_t_* − [*M*]*_t_*υ). The equation can then be re-written as a polynomial equation (Equation 39).

(36) $$0=\left({\left[X\right]}_{t}-{\left[M\right]}_{t}\nu \right){\left(N-l\nu \right)}^{l}-k\nu {\left(N-\left(l-1\right)\nu \right)}^{l-1}$$

Knowing the ligand and macromolecule concentrations, it is possible to solve Equation 39 assuming values of *N*, *l* and *k* to provide a value of *ν* for each injection (*i*). The value of *ν*, can then be used in non-linear regression to extract optimal values for *N*, *l*, *k* and Δ*H* from the heat effect associated with each injection (*q_i_*), given in Equation 37.

(37) $$q_{i}=V_{0}\Delta H\left({\left[M\right]}_{t,i}\nu_{i}-\left(1-\frac{\upsilon }{V_{0}}\right){\left[M\right]}_{t,i-1}v_{i}-1\right)$$

where υ is the injection volume.

Introduction of a cooperativity factor, *α*, results in a more complex closed form of the Scatchard representation (Equation 38). The shape of the resulting Scatchard plot is affected both by the entropic resistance to saturation and the cooperativity parameter, *α*. It is only linear if the apparent negative cooperativity due to the entropic resistance to saturation is compensated by real positive cooperativity.

$$\frac{\nu }{\left[X\right]}=\frac{N-lv}{k}{\left(\frac{\left(2\alpha -1\right)\left(N-lv\right)+\nu -R}{2\left(\alpha -1\right)\left(N-lv\right)}\right)}^{l-1}{\left(\frac{N-\left(l+1\right)\nu +R}{2\left(N-l\nu \right)}\right)}^{2}$$

where:

(38) $$R=\sqrt{{\left(N-\left(l+1\right)\nu \right)}^{2}+4\alpha \nu \left(N-l\nu \right)}$$

Again the equation can be written as a polynomial equation with [*X*] expressed in terms of the total concentration of macromolecule and ligand. This equation can be solved to obtain the total binding parameter assuming values of *N*, *l*, *k* and *α*.

(39) $$\begin{array}{l} 0=\left({\left[X\right]}_{t}-{\left[M\right]}_{t}\nu \right)\left(N-l\nu \right){\left(\left(2\alpha -1\right)\left(N-l\nu \right)+\nu -R\right)}^{l-1}{\left(N-\left(l+1\right)\nu +R\right)}^{2}\\ \;\;\;\;\;\;\;\;\;\;-k\nu {\left(2\left(\alpha -1\right)\left(N-l\nu \right)\right)}^{l-1}{\left(2\left(N-l\nu \right)\right)}^{2} \end{array}$$

The heat exchange associated with each injection can be evaluated using Equation 40, where Δ*h* is the enthalpy associated with the interaction between two adjacent bound ligands.

(40) $$\begin{array}{l} q_{i}=V_{0}(\Delta H\left({\left[M\right]}_{t,i}\nu_{isol,i}-\left(1-\frac{\upsilon }{V_{0}}\right){\left[M\right]}_{t,i-1}\nu_{isol,i-1}\right)\\ \;\;\;\;\;\;\;\;\;+\left(\Delta H+\frac{\Delta h}{2}\right)\left({\left[M\right]}_{t,i}\nu_{sc,i}-\left(1-\frac{\upsilon }{V_{0}}\right){\left[M\right]}_{t,i-1}\nu_{sc,i-1}\right)\\ \;\;\;\;\;\;\;\;\;\;+\left(\Delta H+\Delta h\right)\left({\left[M\right]}_{t,i}\nu_{dc,i}-\left(1-\frac{\upsilon }{V_{0}}\right){\left[M\right]}_{t,i-1}\nu_{dc,i-1}\right))\; \end{array}$$

The total binding parameter is actually the summation of the partial binding numbers (υ*_isol_*, υ*_sc_* and υ*_dc_*, Equation 41 for each of the possible binding modes that a ligand can adopt on a mono-dimensional lattice (see Figure 3):

(41) $$\nu =\nu_{isol}+\nu_{sc}+\nu_{dc}$$

where:

(42) $$\nu_{isol}=\left({\left[X\right]}_{t}-{\left[M\right]}_{t}\nu \right)\frac{N-l\nu }{k}{\left(\frac{\left(2\alpha -1\right)\left(N-l\nu \right)+\nu -R}{2\left(\alpha -1\right)\left(N-lv\right)}\right)}^{l+1}$$

(43) $$\nu_{sc}=\left({\left[X\right]}_{t}-{\left[M\right]}_{t}\nu \right)\frac{\alpha }{\alpha -1}\frac{\left(l-1\right)\nu -N+R}{k}{\left(\frac{\left(2\alpha -1\right)\left(N-l\nu \right)+\nu -R}{2\left(\alpha -1\right)\left(N-l\nu \right)}\right)}^{l}$$

(44) $$\nu_{dc}=\left({\left[X\right]}_{t}-{\left[M\right]}_{t}\nu \right){\left(\frac{\alpha }{2\left(\alpha -1\right)}\right)}^{2}\left(\frac{{\left(\left(l-1\right)\nu -N+R\right)}^{2}}{k\left(N-l\nu \right)}\right){\left(\frac{\left(2\alpha -1\right)\left(N-l\nu \right)+\nu -R}{2\left(\alpha -1\right)\left(N-l\nu \right)}\right)}^{l-1}$$

The nature of the cooperativity dictates how the binding modes change during the course of the titration. In a non-cooperative system, isolated ligands will bind initially, followed by singly contiguous ligands and finally doubly contiguous ligands with two nearest neighbours. In contrast, in a positively cooperative system, the ligands will immediately cluster forming doubly contiguous ligands. The opposite would happen in a system regulated by negative cooperativity. Isolated ligands would form initially, and only when ligand accumulated would singly contiguous ligands be observed. Ligands with two neighbours would only accumulate at very high ligand concentration.

As mentioned previously, reverse titrations can be used to fully characterise the binding isotherms. The same applies for the binding of ligands to lattice-like macromolecules. The binding polynomials used are the same, but the roles of the ligand and macromolecule reversed.

An alternative method of implementing the noncooperative McGhee–von Hippel model in the analysis of ITC data has been proposed by Shriver and coworkers [37], in which the heat observed by each injection is evaluated by

(45) $$q_{i}=V_{0}\Delta H\Delta {\left[X\right]}_{b,i}+h$$

where Δ[*X*]*_b,i_* is the change in concentration of bound protein as the result of the *i*th injection and *h* is the heat of dilution observed with each injection after saturation of the binding sites at the end of titration [38]. The change in the concentration of bound protein is given by

(46) $$\Delta {\left[X\right]}_{b,i}={\left[X\right]}_{b,i}-{\left[X\right]}_{b,i-1}+0.5\frac{\upsilon }{V_{0}}\left({\left[X\right]}_{b,i}+{\left[X\right]}_{b,i-1}\right)$$

The concentration of bound protein, [*X*]*_b,i_*, is given by Equation 47, which can be solved for values of *k* and *l*.

(47) $$0=k\left(1-l\frac{{\left[X\right]}_{b}}{{\left[M\right]}_{t}}\right){\left(\frac{1-l\frac{{\left[X\right]}_{b}}{{\left[M\right]}_{t}}}{1-\left(l-1\right)\frac{{\left[X\right]}_{b}}{{\left[M\right]}_{t}}}\right)}^{l-1}-\left(\frac{\frac{{\left[X\right]}_{b}}{{\left[M\right]}_{t}}}{{\left[X\right]}_{t}-{\left[X\right]}_{b}}\right)$$

### 10.1. Example: Chromatin Protein Sac7d Binding to DNA

Sac7d is a 7 kDa chromatin protein from the hyperthermophile *Sulfolobus acidocaldarius*. It binds non-cooperatively and non-specifically to the minor groove of duplex DNA and is known to induce a significant kink (66°) in the DNA structure [39]. Therefore, to obtain full thermodynamic characterisation of the interaction, titrations were performed between Sac7d and poly(dGdC) and the ITC data were fit to the non-cooperative McGhee–von Hippel model using the method described above [37]. At 25 °C, Sac7d was shown to bind with moderate intrinsic affinity (approximately 833 nM) and a ligand footprint of 4.3 base pairs. The thermodynamic parameters showed that the interaction was driven entirely by entropy (17.5 kcal mol^−1^), with a unfavourable enthalpic contribution (9.2 kcal mol^−1^). The role of entropy in the interaction was attributed to the polyelectrolyte effect which is known to play an important role in promoting binding to DNA. The unfavourable enthalpy was attributed to the energy needed to distort DNA, which is generally believed to be considerable [40]. Kinking is associated with base-pair unstacking, unwinding and bending, which leads to widening of the minor groove as well the release of water and counterions (which would contribute to the favourable entropy term) due to backbone charge redistribution.

## 11. Global Analysis

Recently, global analysis of ITC data has become increasingly popular as it allows multiple titrations, such as normal and reverse titrations, to be compiled into a single dataset from which the thermodynamic parameters can be calculated [41, 42]. It can be expected that global analysis of several datasets (and replicates thereof) will increase the information and precision of the parameters as long as unrecognised systematic errors are not introduced [43]. The applicability of global analysis to multiple ITC datasets has been demonstrated by examining the relationship between temperature and pH dependence on the thermodynamic parameters [44].

However, global analysis departs with the floating parameter *n*, which incorporates both the reaction stoichiometry and concentration errors, meaning *n* is often a non-integer. In global analysis only integral values reflecting the stoichiometry are permitted. Therefore it is necessary to accurately determine the stoichiometry prior to global analysis by alternative biophysical techniques. Concentration errors and incompetent protein fractions are accounted for with a separate error term [45].

A protocol for the global analysis of ITC data for multisite and cooperative binding using the *SEDPHAT* software package has been described by Houtman *et al.* [45]. *SEDPHAT* is a widely used platform for global analysis of data from a variety of biophysical techniques [46].

### 11.1. Example: LAT, Grb2 and Sos1 Ternary Complex Assembly

Global analysis of ITC data was used to examine the role of cooperativity in the assembly of a three-component multiprotein complex involved in signal transduction after T-cell receptor (TCR) activation [45]. The complex comprises proteins LAT, Grb2 and Sos1. LAT is an adaptor protein, which is rapidly phosphorylated after TCR activation [47]. Grb2 can bind multivalently to LAT phosphopeptides that contain two or more SH2 domain binding sites at tyrosines at positions 171, 191 and 276 [48]. Sos1 has two binding sites for Grb2 with significantly different binding affinities [48]. To reduce the complexity of the system, oligomerisation was eliminated by truncation of Sos1 to an *N*-terminal fragment comprising a single Grb1 binding site (Sos1NT). Similarly, a single phosphorylated LAT peptide was used. Thus, LAT*^pY^* ^191^ can bind one Grb2 molecule, which in turn can bind one Sos1NT molecule.

Two titrations were performed: LAT*^pY^* ^191^ into Grb2 alone and LAT*^pY^* ^191^ into a stoichiometrically mixed 1:1 Grb2-Sos1NT solution. The best fit parameters for the binding of LAT phosphopeptide to Grb2 from a global model for the ternary interaction were *K_d_* = 286 nM, Δ*G* = −8.9 kcal mol^−1^ and Δ*H* = −3.9 kcal mol^−1^. In the presence of Sos1NT, the cooperativity factor (*α*) was calculated as being 0.54. The presence of cooperativity corresponded to a Δ*g* of 0.37 kcal mol^−1^ and Δ*h* of −3.9 kcal mol^−1^. A model without permitting cooperativity was unable to account for systematic difference in the initial heats of injection for LAT phosphopeptide to Grb2 in the presence and absence of Sos1NT and resulted in an almost threefold increase in the *χ*^2^ of the fit.

## 12. Combination of ITC and NMR to Study Cooperativity

NMR spectroscopy is one of the few experimental techniques capable of measuring the occupancies of individual binding sites on proteins and therefore determining the microscopic binding affinities. Coupling this site-specific data with the macroscopic binding data from ITC allows a complete description of the binding properties of the system. A method of determining cooperativity using NMR spectroscopy has been described using the isotope-enriched two-dimensional heteronuclear single-quantum coherence experiment (2D HSQC) [26]. The ligands are isotopically labeled (usually ^1^H and ^13^C or ^15^N) whilst the macromolecule remains unenriched. Spectra are recorded at different molar ratios and the peak volumes are integrated. Isotherms are generated by plotting the peak volume integration against molar ratio. The data is then fitted to site-specific binding models to obtain the thermodynamic parameters.

### 12.1. Example: Glycocholate Binding to I-BABP

Human ileal bile acid binding protein (I-BABP) has two binding sites for glycocholate, the physiologically most abundant bile salt [49]. The binding sites have intrinsically weak affinity for glycocholate, but extremely strong positive cooperativity. The intrinsically low affinity means that at low ligand–protein ratios a significant amount of glycocholate remains unbound, however at high ligand–protein ratios the positive cooperativity ensures more ligand is bound.

The cooperativity in this system was analysed using ITC and heteronuclear 2D HSQC NMR spectroscopy [26]. The calorimetry data were fit a sequential model, but did not contain enough information to define the enthalpy terms accurately. For the NMR experiment, glycocholate was isotopically labeled with ^1^H-^15^N; I-BABP was not labeled. ^1^H-^15^N spectrum were recorded at different molar ratios. In each spectrum three main resonance peaks were observed, corresponding to unbound glycocholate, glycocholate bound at site 1 and glycocholate bound at site 2. Binding curves for each site were generated by plotting the peak volume integrations against molar ratio. The NMR curves were then fitted using a site-specific binding model, as described by Tochtrop *et al.* [26].

From the NMR binding curves, the microscopic affinities were calculated as 1.5 mM for site 1 and 2.1 mM for site 2. The cooperativity constant, *α* was calculated as 860 yielding step-wise dissociation constants of 1.8 mM for binding of the first glycocholate molecule and 1.5 *μ*M for the second interaction. Thus, I-BABP displays extreme positive cooperativity for the binding of its substrate.

Whilst the example illustrates cooperativity between two sites, the experiment can be extended to systems with multiple sites as long as NMR peaks corresponding to each site can be resolved. Also, by using unique isotope enrichment for different ligands, it is possible to study systems with multiple ligands [26].

## 13. Conclusions

ITC provides information rich data with kinetic, thermodynamic and stoichiometric parameters defined in a single experiment. As one of the few biophysical techniques able to dissect the thermodynamics of a binding event, it has rapidly become an important tool in the study of biological interactions, including those involved in ternary complex formation and the binding of multivalent ligands. Assemblies of such systems are often regulated by cooperativity. Cooperativity is best understood in terms of thermodynamics, as not all cooperative systems undergo conformational changes that are often associated with allosteric modulation. Thus, ITC is the ideal technique to ascertain the origin and underlying mechanisms of cooperativity.

To obtain full thermodynamic characterisation of the cooperativity, multiple complementary titrations are often necessary to define binding and cooperativity parameters. The importance of correct experimental design and selection of suitable binding models cannot be understated. The recent application of model-independent binding polynomial formalism to analyse ITC data should reduce the number of examples in the literature where incorrect or insufficient binding models are used.

The example of glycocholate binding to I-BABP illustrates the abundant information that can be obtained from the combination of ITC with other biophysical techniques (full characterisation of the microscopic and macroscopic binding affinities). Global analysis offers the possibility of combining ITC with data from other techniques such as surface plasmon resonance [50], NMR [51], circular dichroism [52], spectrofluorometry [53] and dynamic light scattering [50].

## Figures and Tables

**Figure 4.**
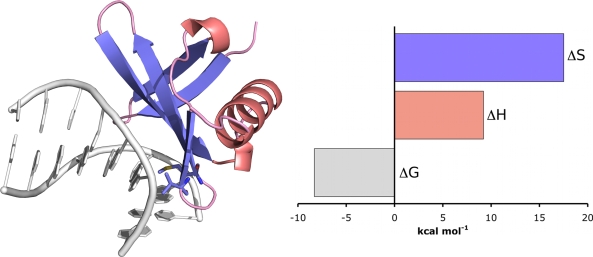
The crystal structure of chromatin protein Sac7d bound to a nucleic acid fragment (PDB ID: 1AZP) demonstrates that binding induces a 66° kink in the structure of DNA. Thermodynamics of the binding event determined by application of the non-cooperative McGhee–von Hippel model to ITC data demonstrates that binding is characterised by a favourable entropy term and an unfavourable enthalpy term attributed to the energetic penalty of kinking DNA.

**Table 1.** A macromolecule with two binding sites is capable of existing in three states: unbound, singly bound or doubly bound. The relative concentration of these states depends on whether the macromolecular binding sites are identical and whether they are independent. The binding polynomial for each model is obtained by the summation of the terms in each column.

Binding state	General	Identical independent	Nonidentical independent	Cooperative
Unbound	1	1	1	1
Singly bound	*β*_1_[*X*]	2*k*[*X*]	*k*_1_[*X*] + *k*_2_[*X*]	2*k*[*X*]
Doubly bound	*β*_2_[*X*]^2^	*k*^2^[*X*]^2^	*k*_1_*k*_2_[*X*]^2^	*αk*^2^[*X*]^2^
